# From co-creation to technical bias detection methods: an interdisciplinary showcase from the BIAS project

**DOI:** 10.3389/frai.2026.1795293

**Published:** 2026-07-06

**Authors:** Mascha Kurpicz-Briki, Catherine Ikae, Alexandre Puttick, Guðbjörg Linda Rafnsdóttir, Ragna Kemp Haraldsdóttir, Carlotta Rigotti, Eduard Fosch-Villaronga, Maria Sangiuliano, Mark W. Kharas, Roger A. Søraa

**Affiliations:** 1 School of Engineering and Computer Science, Bern University of Applied Sciences BFH, Biel/Bienne, Switzerland; 2 Faculty of Sociology, Anthropology and Folkloristics, University of Iceland, Reykjavik, Iceland; 3 eLaw—Center for Law and Digital Technologies, Leiden University, Leiden, Netherlands; 4 SmartVenice, Venice, Italy; 5 Department of Interdisciplinary Studies of Culture, Norwegian University of Science and Technology NTNU, Trondheim, Norway

**Keywords:** bias detection, biases, cultural nuances, interdisciplinary methods, languages, machine learning, natural language processing, societal stereotypes

## Abstract

Societal stereotypes are often reflected in, and can be reinforced by, machine learning models and linguistic resources such as word embeddings. While various benchmarks and bias detection methods have been proposed, most focus exclusively on English. When applied to other languages, these approaches typically rely on direct translations of English resources, overlooking language- and culture-specific nuances. In this paper, we introduce BIAS-WEAT and BIAS-SEAT, two novel metrics designed to detect biases in word embeddings and language models for Dutch, German, Icelandic, Italian, Norwegian and Turkish. Drawing on real-world biases identified through co-creation workshops with native speakers in the context of a hiring situation, we translated these insights into technical evaluation metrics that are applicable to general-purpose language resources. Our interdisciplinary study demonstrates how language models embed and reproduce biases that are specific to their linguistic and geographic contexts, underscoring the need for culturally grounded approaches to bias detection.^1^

## 1 Introduction

Machine learning models trained on biased data risk reproducing and amplifying existing social inequalities. These models tend to privilege Western English-speaking contexts while demonstrating reduced reliability in low-resource languages (Qu and Wang, 2024). This English-centric orientation constrains global applicability, reinforces stereotypes derived from non-representative training sources, and limits evaluation in more complex decision-making domains. Current approaches to bias detection remain disproportionately focused on English, leaving other linguistic and sociocultural contexts underexamined (Franklin et al., 2024; Raj et al., 2024).

The data that underlie machine learning models often reflect historical inequities, perpetuating systemic disadvantages and strengthening harmful stereotypes (Gichoya et al., 2023; Njoto et al., 2024). Such biases conflict with ethical principles of fairness and inclusion and may undermine organizational performance when embedded in workplace settings. In recruitment contexts, social costs are borne primarily by job applicants, who often have weaker information and bargaining power (Rigotti and Fosch-Villaronga, 2024), but in other applications of AI these costs may manifest differently, potentially affecting different groups or aspects of social and economic participation. Furthermore, reliance on predominantly English training corpora means that these models have lower capabilities in smaller or low-resource languages, where limited written material, online presence, and language-specific tools restrict effectiveness (Pava et al., 2025). Addressing these limitations is critical for advancing the reliability, fairness, and cross-cultural applicability of machine learning models.

In this context, there remains a pressing need for systematic investigations of bias in diverse language and sociocultural contexts. While these limitations are often framed as a matter of language coverage or data availability, they also point to a deeper methodological challenge: bias detection methods typically rely on predefined categories and assumptions about what constitutes socially relevant harm. These assumptions are rarely interrogated and are often shaped by dominant, Anglophone sociocultural contexts. As a result, even when bias detection tools are extended to additional languages, they risk reproducing externally imposed definitions of bias that may fail to capture locally salient forms of discrimination or exclusion.

Because such forms of harm are socially situated, institutionally mediated, and often contested, they cannot be adequately specified through translation or expert-driven modeling alone. This raises a critical question for bias research: how can technical bias detection methods meaningfully reflect real-world inequalities across diverse linguistic and sociocultural settings, rather than merely translating existing tests? Our study addresses this challenge by grounding multilingual bias detection in co-created, context-sensitive definitions of real-world bias, developed through interdisciplinary collaboration and subsequently operationalized within technical evaluation methods applied beyond English.

This methodological challenge is particularly visible in the bias detection resources and evaluation practices developed within the field of Natural Language Processing (NLP). Societal stereotypes are reflected in language models and word embeddings (Caliskan et al., 2017; May et al., 2019). Various approaches have been developed to detect (May et al., 2019; Kurita et al., 2019) and mitigate such biases, as well as benchmark datasets that allow for their systematic evaluation (Nangia et al., 2020; Parrish et al., 2022). However, most of this research has been carried out on English data, a limitation that has been widely recognized in NLP research (Joshi et al., 2020; Søgaard, 2022). A critical survey by Blodgett et al. (2020) examined 146 articles addressing bias in NLP systems, finding that nearly all focus exclusively on English. More recent reviews confirm this imbalance: Ramesh et al. (2023) emphasize that fairness research in NLP remains overwhelmingly English-centric, while Gamboa et al. (2025) identified only 106 studies targeting bias in multilingual or non-English pretrained language models. However, the state-of-the-art in this field typically concentrates on translating or slightly adapting existing tests to measure bias. For example, Mukherjee et al. (2023) extended the WEAT tests to 24 languages (WEAThub). They, however, mention that they excluded tests involving target or attribute words specific to European American or African-American names, as they lack relevance for non-Western and non-European languages and can thus not be translated (such tests can, however, be adapted, as described later). A similar approach was chosen by Lauscher and Glavaš (2019). In our own previous work (e.g., Kurpicz-Briki, 2020), we adapted these names based on statistical information of the relevant language region (e.g., the most common male and female names in Switzerland). The SEAT framework May et al. (2019) was adapted from the original English version by translating or localizing the target and attribute word lists and, where necessary, modifying the sentence templates to better fit the grammar and sociocultural norms of each language. For German, Rankwiler and Kurpicz-Briki (2024) created German equivalents of the original WEAT and SEAT word pairs (e.g., gendered occupations and names) and validated them through native speaker judgments. In Maltese, Galea and Borg (2025) combined direct translations with culturally appropriate replacements to reflect local naming conventions and gendered terms, applying SEAT to both monolingual and multilingual Maltese models. For Italian, Puttick and Kurpicz-Briki (2025) extended SEAT with semantically bleached templates and intersectional attribute sets (e.g., gender-profession, gender-religion) to test multiple dimensions of bias in Italian contextual embeddings. Together, these multilingual adaptations demonstrate that representational biases are not limited to English models but persist across diverse linguistic and cultural settings.

In this paper, we therefore adopt a bottom-up, co-creative approach to bias detection, starting from real-world manifestations of discrimination and tracing how these are reflected in word embeddings and language models. This approach is operationalized through the interdisciplinary Horizon Europe project BIAS,^2^ which brought together researchers from the fields of computer science, law, social sciences and humanities to collaboratively identify socially salient forms of bias across different European contexts. Based on these co-creation processes, we introduce two novel bias detection metrics—BIAS-WEAT and BIAS-SEAT—which transfer empirically grounded, context-sensitive bias definitions into technical evaluation frameworks, with a particular focus on European and low-resource languages. This allows to address bias detection and mitigation for languages so far underrepresented in the research. Additionally, we propose a blueprint for future projects to build context-sensitive and culturally adapted datasets for this purpose making use of co-creation activities.

This paper is structured as follows: In Section 2, we present the materials and methods, showcasing show the novel metrics were derived based on co-creation activities. We also present the novel metrics—BIAS-WEAT and BIAS-SEAT—that detect bias in different European languages. In Section 3, we then discuss the results, after having applied BIAS-WEAT and BIAS-SEAT to different word embeddings and language models in German, Dutch, Norwegian, Icelandic and Turkish. Finally, we discuss the results in Section 4 and bring them in relation to the interdisciplinary field of literature.

The code and data presented in this paper are available in the project's Github repository.^3^

## 2 Materials and methods

### 2.1 Overall setup

**Figure 1:**
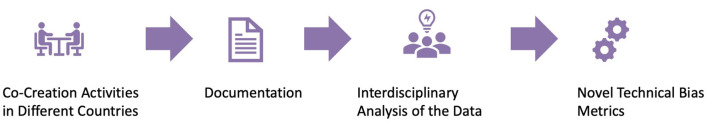
Workflow of the BIAS Human Engagement for Resource Creation process: transforming human inputs about real-world biases from co-creation to bias detection metrics for word embeddings and language models.

The creation of the novel bias metrics BIAS-WEAT and BIAS-SEAT presented in this paper based on the real-world biases from the co-creation workshops happened in an iterative process, as shown in Figure 1.

In a first step, co-creation activities were conducted in the different partner countries of the BIAS consortium. One aim of the workshop was to identify specific biases that might be encountered in the labor market in these regions, and how these biases were expressed in the corresponding languages. For example, specific sensitive attributes might be seen differently in a German application process in Switzerland, compared to an application process in Turkish in Türkyie. The methodology of these co-creation workshops are described in Section 2.2. The discussions at the co-creation workshops were then carefully documented, and corresponding expressions indicating biases were extracted. This step of data analysis was done in close collaboration between the technical researchers and the social science and humanities researchers as well as legal scholars in the project. These insights were then transformed into dedicated novel bias detection metrics for the different languages, as described in Section 2.3.

### 2.2 Co-creation as method: generating intersectional lexical data for bias analysis

The BIAS project adopts a participatory and co-creative approach to define the requirements for identifying and mitigating bias in AI systems. In BIAS, the co-creation workshops serve the purpose of providing input for the technological development, providing AI developers with insights into real-world experiences related to bias detection and mitigation.

The value of adopting a multi-stakeholder approach in the design of AI solutions has been well-documented in relevant literature, and it is part of the overall feminist/intersectional and socio-technical theoretical framework featuring the project (Wajcman, 2009; Costanza-Chock, 2020). It has been widely recognized that designing these solutions entails not only a multidisciplinary technological effort but also benefits from incorporating ethical, societal, political, legal concerns and frameworks (Leikas et al., 2019) that make a multi-stakeholder approach necessary.

Seven workshops^4^ were organized in Estonia, Iceland, Italy, the Netherlands, Norway, Switzerland, Türkiye, between June and July 2023, engaging 144 participants overall: HR officers/managers (29 people and 7 people representing HR networks), workers (33), members of NGOs and CSOs addressing discriminations (32) and AI specialists (26). Additional stakeholders included employer associations, regional inclusion policymakers, researchers, and academics. Women represented 66.67% of participants. During the workshop, participants were asked to simulate an early recruitment process, which involved evaluating candidates' CV as personas and drafting their motivation letters in response to job vacancies announced by HR managers using these as “scenarios”, and a set of pre-configured “personas” or fictional characters that each partner could adapt starting from some visible/pre-set features including gender and race/ethnicity, and other elements related to soft-skills and hobbies that were aimed at triggering potential bias perceptions or challenging them (i.e. a cisgender man with a knitting hobby), in view of stimulating discussions. The workshops employed a structured co-creation methodology to generate and identify wordlists capturing bias-prone linguistic associations in real-life recruitment contexts. The four proposed scenarios included Job Offers descriptions from different companies/organizations in a variety of sectors: an iron/steel industrial company, a research institute, a tech company, a private school. Participants were assigned to mixed-expertise groups (HR officers, AI specialists, civil society representatives) and worked with a common job-offer scenario reflecting sectoral patterns of gendered and racialised segregation. Each group received a distinct “persona”—a fictional applicant profile combining selected gender and race/ethnicity categories, with options to include non-binary identities and locally relevant minority groups. Personas were deliberately designed with both positive and negative connotations to support the construction of balanced lexical sets suitable for subsequent bias-detection in word embeddings.

The group work followed a sequence of tasks intended to elicit naturalistic linguistic production and critical reflection. First, participants (excluding the HR officer) collaboratively drafted a cover letter from the viewpoint of their assigned persona. This exercise generated a primary corpus of vocabulary, descriptors, and narrative elements intuitively associated with specific intersectional profiles. Second, the cover letter was collectively analyzed to identify words, expressions, and semantic associations that could trigger positive or negative biases related to gender, race/ethnicity, or other social dimensions. Discussions focused particularly on how otherwise neutral terms acquire bias-laden connotations when linked to certain identity markers or life circumstances. Rapporteurs documented all lexical items and rationales in detail.

In a subsequent step, groups rephrased sections of the cover letter identified as problematic. The juxtaposition of original and revised formulations enabled further clarification of which linguistic features were perceived as bias-inducing. All workshop outputs–draft and revised cover letters, annotated sticky notes, and rapporteur reports–formed the empirical basis for extracting structured wordlists and categories of biased associations. These human-generated wordlists constitute the main outcome of the exercise and the foundational input for developing tools measuring bias in word embeddings. From the 7 workshops' reports (all in English), 7 spreadsheet/matrix with the “biased” words in the native languages and including sub-sheets for each different individual personas' profile chosen were elaborated. In these sub-sheets, the word/attributes/sentences within the various dimensions were further categorized as: (1) potentially bias-generating or (2) controversial (if no agreement is found in the group).

Re-formulated versions of each word from the spreadsheet that attempted to avoid or mitigate bias were also included. For potentially bias-generating and controversial words, partners were encouraged to report full sentences and to add comments for more in-depth, contextualized interpretations.

### 2.3 The BIAS human engagement for resource creation process

#### 2.3.1 Overview

Across all workshops, the research teams collected 389 words and sentences potentially associated with bias in recruitment. As participants were asked to classify each term as eliciting a positive or negative bias, the distribution of classifications was found to be nearly symmetrical: 181 items were linked to positive bias and 182 to negative bias, while 26 items were marked as “context-dependent”. An additional 59 items were labeled “controversial,” reflecting disagreement on their bias orientation, and 58 items were rephrased to search for bias-mitigating terms.

The data was analyzed using a process that was jointly developed within the interdisciplinary consortium, through close collaboration between technical researchers, native speakers, and scholars from the social sciences and humanities (SSH).

To develop the novel bias detection metrics, the concepts that were mentioned at the co-creation workshops were used as source for the word lists in the style of existing bias metrics (in particular WEAT (Caliskan et al., 2017)), and, based on that, the sentence lists for the bias metrics for contextualized embeddings [following the SEAT (May et al., 2019) format]. When analyzing the word lists and notes generated in the workshop editions in the different languages and locations, topics of discussion (e.g., specific biases that were mentioned) were picked up in the analysis process, to develop the bias detection methods.

In some cases, very concrete discussions about bias against specific groups came up in the workshop (e.g., in Icelandic referring to *Asians being potentially technology nerds*). In other cases, it was more challenging to directly extract the specific biases based on the notes. In these cases, the targets (= group potentially being subject to discrimination) were identified based on the co-creation workshops and combined with literature searches and discussions with native speakers, as specific words for the two target and attribute (= associated concepts or sentiments for a given target group) dimensions are necessary for this technical task.

Whereas sometimes concepts have been expanded, in other cases specific words from the co-creation workshop or their synonyms were taken as seed words (i.e., the original words of bias building the foundation for our novel methods for bias detection). The word lists were discussed with native speakers of the respective languages, ensuring a good understanding of the outcomes of the co-creation workshops.

The resulting datasets are available in the Github repository.^5^ The following subsections describe the dataset creation with more detail.

#### 2.3.2 Metric 1: BIAS-WEAT

As a first step, the BIAS-WEAT metric was developed. This method is based on the structure of the well-known WEAT metrics (Caliskan et al., 2017) and is composed of different tests: each test requires two sets of target words (e.g., local and foreign first names), and two sets of attribute words (e.g., positive and negative adjectives). Such methods can be applied to static word embeddings, as for example *fasttext* embeddings (Bojanowski et al., 2016). These tests were deducted from biases that were mentioned in the discussions in the co-creation activities, in order to measure to what extend the societal biases are reflected in the word embeddings.

**Figure 2:**
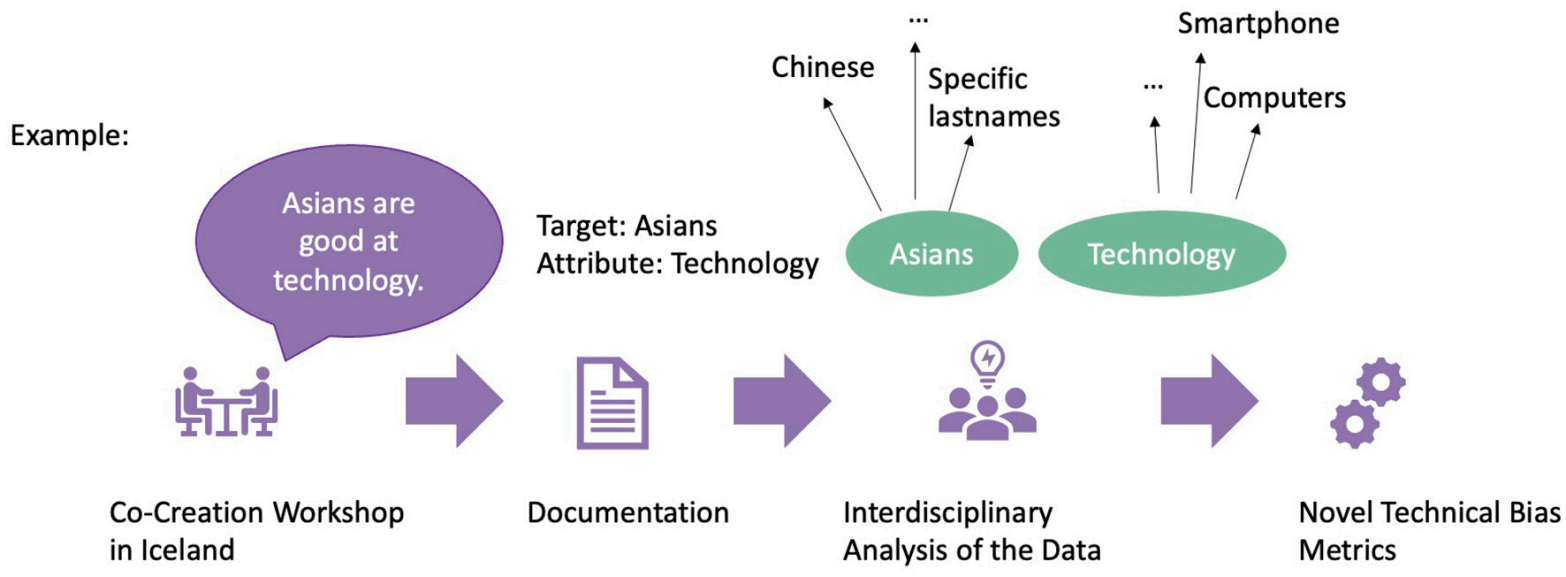
A concrete example from the Icelandic co-creation workshop. Note that the workshop was conducted in Icelandic and the words have been translated here for better readability.

This process is illustrated with a concrete example from the Icelandic workshop in Figure 2, based on the example sentence mentioned previously.

(1) The target group *Asians* was extracted from the co-creation materials, with the attribute *Technology*.

(2) In interdisciplinary discussions and under consideration of the relevant literature and local statistics, other ways of expressing this concept were explored. For example, being *Asian* could be indicated by having a specific last name or by being of a specific nationality, for example, *Chinese*.

(3) Similarly, the concept of *Technology* can be composed of different aspects, for example smartphones and computers.

Using this method, the bias detection metric BIAS-WEAT for Icelandic was derived from the co-creation workshop results. As explained earlier, this metric is composed of sets of two groups of target words and two groups of attribute words that are then evaluated against each other.

The number of different forms of bias that could be identified based on the notes of the workshops was very different between the different countries. Depending on how the conversations in the co-creation workshops evolved (e.g., variety and topics of discussion), in some cases the analysis of the workshop results was rather challenging (e.g., Dutch), in other cases not all the mentioned concepts could be fully explored yet (e.g., Icelandic) and leave additional room for future research later in the project by exploring the additional words that were mentioned. It was primarily dependent on the participants and conversational dynamics experienced throughout the workshops. A more detailed description of the procedure for each language will follow along with the presentation of the novel bias metrics in a later section.

#### 2.3.3 Metric 2: BIAS-SEAT

The word lists described in the previous subsection are suitable for static word embeddings, as for example *fasttext* (Bojanowski et al., 2016) available in many languages. However, these static word embeddings have the limitation that they do not consider context. For example, the word *Jaguar* referring to the animal, and the word *Jaguar* referring to the car have the same embedding. Overcoming this limitation, contextualized word embeddings such as those generated with transformer-based models (Vaswani et al., 2017) are more powerful for many NLP tasks.

Accordingly, an additional bias detection metric - BIAS-SEAT - was developed to measure bias in these kinds of models. Instead of testing single words as in BIAS-WEAT, following the structure of the WEAT tests (Caliskan et al., 2017), we here rely on methods for contextualized word embeddings that rely on entire sentences.

We develop our novel method BIAS-SEAT based on the solid foundations of existing work that was done for English, using the structure of the SEAT method (May et al., 2019). The idea is to construct simple sentences around the potentially biased words. For example, sentence templates as the following are used for nouns:

*This is a WORD*.
*A WORD is here*.
*A WORD is there*.

In our case, the placeholder *WORD* can then be filled with the different words from the BIAS-WEAT tests. In English, such structures can be used easily with different words; one may simply need to adapt the article *a* to *an* in some cases. Such an adaptation can be much more challenging for other languages, depending on their morphological and linguistic properties, thus providing additional challenges in the development of the BIAS-SEAT method.

For example, in German, the template *This is a WORD*., with *WORD=friend*, could have different forms for a male or a female friend, and requires an adaptation of the article *a* to the gender of the following word.

*Dies ist ein Freund. Dies ist eine Freundin*.

**Figure 3:**
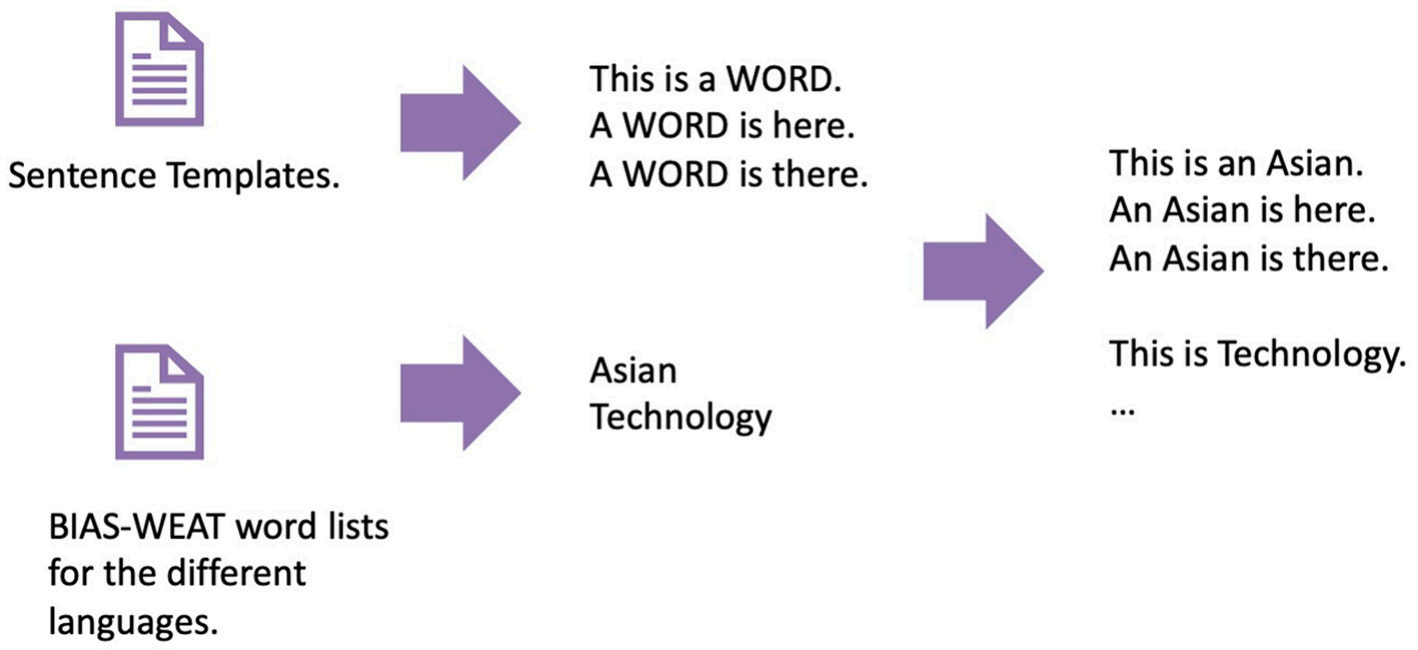
The method on how the BIAS-SEAT metric was developed based on sentence templates and the word lists from BIAS-WEAT described earlier. The example is in English for better readability, however, this process was conducted for the different languages with their specific linguistic challenges as described in the text.

Figure 3 shows the overall procedure in English. In the work presented in this paper, we have executed these steps for Dutch, German, Icelandic, Italian,^6^ Norwegian and Turkish.

At the same time, one of the goals of our sentence generation process for the different languages was automation. An efficient BIAS-SEAT sentence template generation process is required to enable future work to easily create resources for different languages. We therefore explored two different methods to obtain the sentences required for the new metric.

In both cases, two-fold human engagement was involved. We consider this crucial in order to ensure correctness and appropriateness of linguistic resources. Whereas much can be covered by automation, verification is still required, especially for low-resource languages and in sensitive contexts like bias detection. The aim of automation was to reduce these steps as much as possible to a verification process (rather than a manual curation or generation process) and requiring as few manual corrections as possible:

- **Step 1**: Given a BIAS-WEAT word list *W* for a language *A* and a set of templates, generate the sentences for language *A* (e.g., Icelandic or German) and word list *W*, considering the different linguistic challenges.
- **Step 2**: Plausibility verification of the technical collaborator (not native speaker of language *A*), e.g., by manually adapting the number of entries in the lists.^7^
- **Step 3**: Verification of the resulting sentences by a native speaker of the language *A* from the BIAS consortium.
- **Step 4**: Execution of the technical bias test on the different models for language *A*.

We experimented with different implementations of Step 1 to generate the new sentence lists based on the word lists of the co-creation workshops:

- **Step 1 - Method 1**: Scripts based on NLP libraries and grammatical structures of the sentences and specific languages, and
- **Step 1 - Method 2**: Generation of sentences with latest AI models (GPT), using different prompting variants.

We started with Icelandic, Italian and Norwegian and applied a programmatic approach implemented in Python, relying on common NLP resources (e.g., Spacy^8^ models and libraries). For example, the Spacy model for Italian was used to get the type of a word (e.g., noun, verb), whereas exceptions had to be handled manually as a list of expressions. The following line shows the example for adjectives that were not correctly parsed by the library:


  exceptions_adj = [‘‘male'',‘‘trans'',‘‘transgender'',
   ‘‘etero'', ‘‘cis'', ‘‘0cisgender''1, ‘‘2queer''3]


For example, the Italian word *male* (engl: bad, the bad) can be a noun and an adjective, and thus automated processing has to decide for one of the two options. Other words that were not recognized properly were often rarer words or words that have been potentially used more frequently in the recent past.

The programmatic approach then explored further automation based on regular expressions to explore automatically which article needs to be employed, based on grammar rules of Italian. Depending on the grammatical gender (*un, una*) or the starting letter(s) of a word (*un, uno*) different articles need to be placed in the templates to obtain grammatically correct sentences.

Similar programmatic approaches were applied for Norwegian and Icelandic. For example, in Norwegian, the word *Islam* was correctly classified as a noun by the library, however, it did not have a grammatical gender assigned. We considered it as grammatically masculine, as it is of masculine grammatical gender in the Arabic language. This is an example of an exception that was handled in our programmatic approach.

Even though many exceptions could be handled by the scripts, several corrections and feedback loops with the native speakers were still required. Given these challenges, and due to the recent advances in the field of large language models in parallel to our research, a different method was explored for Dutch, German and Turkish.

For example, for Turkish, the following prompt template to GPT-4o was developed to generate the sentences using a large language model:


  prompt = ‘‘''‘‘ Consider the following templates for
  nouns: ''‘‘'' + str(templates_nouns) + ‘‘''‘‘ Consider
  the following templates for adjectives: ''‘‘0''1 +
  str(templates_adjectives) + ‘‘2''3‘‘4
  For each WORD in Turkish that I will provide you,
  fill all the corresponding templates,
  i.e., if a noun, only use the noun templates,
  if an adjective, use the adjective templates.
  Consider: a) plural form must be adapted
  based on grammar rules, b) adapt the sentence
  if needed to be grammatically correct,
  c) adapt capital letters as needed to be
  correct. Do NOT modify the sentence in any
  other way. Don't output anything else, just
  the list of sentences, formatted as in
  the templates. Word: ''5‘‘6''7
 

The sentence templates for the different word types (e.g., *templates_nouns*) were defined before-hand and fed into the prompt as well as the specific words of the BIAS-WEAT word lists. This approach is not the most efficient in terms of number of queries made to OpenAI API (to access the GPT model), however, our preliminary tests revealed that when several sentences were processed with the same request, more deviations from the expected output format were observed. The sentences generated were in most cases evaluated as grammatically correct by the native speakers.

After having generated sentence lists for the different languages, we compared these two main approaches and analyzed their respective advantages and disadvantages. The first, a *programmatic approach using NLP libraries*, provides precise control over language-specific processing and ensures reproducibility through versioned, deterministic code without reliance on external APIs. Its main drawbacks are the need for considerable expertise in language-specific tools and the substantial development effort it entails. The second, an *API-based prompt method using GPT*, is easier to implement and offers immediate multilingual support without additional coding, making it suitable for rapid experimentation. However, this convenience comes with disadvantages, such as potential API costs and reduced reproducibility, as the underlying model versions may change over time.

Even though the sentences generated in the context of the BIAS-SEAT metric with the above methods were mostly grammatically correct, they were often perceived as *not natural* by the native speakers. The native speakers stated in different cases that those sentences would not be naturally used by a native speaker and are thus potentially not representative examples of sentences. Whereas a lot can be achieved by automation of language processing, this statement demonstrates the importance of human language perception in the context of automated language processing, especially when it comes to low-resource languages.

### 2.4 BIAS-WEAT and BIAS-SEAT: novel bias detection metrics

#### 2.4.1 German

The resulting data from the workshop in Switzerland for the German language^9^ was the foundation for the creation of the wordlists CW1-CW5. They cover biases related to gender, hobbies, family status, immigration, and productivity. A topic that appeared in the workshops was the differences between age groups and how this might impact the application process. Different topics were mentioned in relation to the age groups, including passion, curiosity, home office, flexibility and work-life-balance. In the context of the labor market, we grouped these attributes that can be seen as positive or negative into the categories *productive* and *unproductive*, as we need two ideally opposite concepts for the WEAT experiments. These build the foundation for the tests CW1, CW2 and CW5.

The German-language co-creation workshops (held in Switzerland) led to the development of five bias test categories designed to capture stereotypes specific to the German language and cultural context. *CW1* contrasts productivity-related and family-related associations (productive vs. unproductive, career vs. family/children). *CW2* examines the link between productivity and age (productive vs. unproductive, young vs. old). *CW3* explores social belonging and cultural identity (traditionalists vs. communicators, native vs. immigrant). *CW4* targets gendered leisure activities (male vs. female hobbies). Finally, *CW5* combines productivity with gender associations (productive vs. unproductive, male vs. female). Together, these categories form a linguistically grounded test suite for analyzing bias in German word embeddings and language models.

Another topic mentioned in the co-creation workshop was the potential bias toward women and, in particular, whether the woman currently had a family or could potentially have a family in the future. These aspects are confirmed by a recent white paper by the Boston Consulting Group and the EqualVoice initiative.^10^ The study states that there are still significant differences between men's and women's careers in Switzerland. Mothers were approximately three times as likely to look for a part-time job compared to fathers. The study identified a stereotypical family model (still prevalent as a societal norm in Switzerland) of a (female) caregiver and a (male) financial provider or the necessity of to childcare as potential reasons for this difference. We covered these aspects by the tests CW1, CW4 and CW5.

Differences between migrants (including descendants from migrants) and local people were discussed in the co-creation workshop. In Switzerland, in 2023, 41% of the permanent resident population aged 15 or over had a migration background, and 27% had a foreign nationality.^11^ The perception of these two groups is therefore crucial in the context of the labor market and is covered with test CW3, using traditionalism and communication (language) skills as word groups.

Earlier versions of the German wordlist for the term *productive* contained the word *Karriereorientierung* (engl: orientation toward career). This was replaced with the word *Effizienz*, as discussions within the team revealed that the term *Karriereorientierung* might be perceived as naturally related to the words *young* and *career* and thus potentially distort the results.

#### 2.4.2 Dutch

Due to the background of one of the profiles used in the co-creation workshop in the Netherlands, people with disabilities were one target group of our work in this country. This was picked up in the discussions: it was explicitly pointed out that this is “a person with a disability” (“Een persoon met een beperking”). At the same time, it was mentioned that at least 50% of individuals with a disability are unemployed. Additionally, existing work has identified that the bias of believing that people with disabilities are unqualified, unproductive and expensive to hire might hinder employers to employ them (Ameri et al., 2018). This led to a specific WEAT test in Dutch about disability and productivity (CW1_NL).

The co-creation workshop participants raised the concern of potential discrimination regarding one profile being trans-gender. Indeed, the Dutch trans-gender network report Inclusion4All^12^ indicates that this discrimination on the labor market can happen based on “emotive reactions such as disgust” or due to “association with mental or physical illness” [Inclusion4All report based on De Lombaerde et al. (2021)]. Therefore, a specific test regarding the stereotype of the LGBTQIA+ community and psychological stability was developed for Dutch (CW2_NL).

Overall, the co-creation workshop for Dutch resulted in two new bias test categories reflecting locally relevant stereotypes. *CW1_NL* captures associations between disability and productivity, representing perceptions of employability and capability in professional contexts. *CW2_NL* examines implicit links between LGBTQIA+ identity and psychological stability, addressing stereotypes related to mental health and social inclusion. Together, these categories extend the multilingual bias evaluation framework by integrating bias dimensions that are particularly salient in the Dutch sociocultural context.

#### 2.4.3 Norwegian

People of Iranian origin were one target group of our work in Norwegian due to one of the personas in the Norwegian co-creation workshop having an Iranian background. There are different ways for how this origin can be visible, including, for example, a person's name indicating nationality. A study (Midtbøen and Rogstad, 2012) has investigated whether ethnic discrimination occurs in hiring processes in the Norwegian labor market, and explores potential explanations for discriminatory practices. They investigated the matter by sending out 1800 paired, fictitious job applications to real job advertisements. Each two applications were identical regarding language, education and work experience, but differed from each other with the applicants names signaling being from the Norwegian ethnic majority and other minority backgrounds respectively. In a second step, in depth interviews were conducted with a selection of employers. The results have shown that a substantial barrier to access to employment market for people with ethnic minority background exists. The likelihood of being called in for a job interview was reduced on average by about 25 percent for applicants with foreign-sounding names compared to identically qualified applicants with a Norwegian majority background. These findings have been further supported in other papers (Quillian et al., 2017, 2019). Similarly, Carlsson et al. (2021) have also researched gender discrimination in the Nordic region, finding that persistent gender disparities in top-level academic positions.

We selected in our tests typically Norwegian first names,^13^ and common first names of the selected minority group from Iran, following a similar method as used in Kurpicz-Briki (2020).^14^ We have assessed positive and negative sentiment in NO1 (as in the existing WEAT test from Caliskan et al. (2017) for African-American and European-American names), and potential differences between the concepts of success and failure in NO4 (following Kurpicz-Briki and Leoni, 2021). Additionally, it was mentioned during the workshop that “Iranians are good at selling” (*Iranere er gode på salg*). Based on that, the test NO7 was defined.

The study described in the previous paragraphs (Midtbøen and Rogstad, 2012) cites one interviewee that is concerned about what his customers think if he hires a (presumably) Muslim man. Another study (Nessa, 2024) cites different studies that argue that hostility, prejudice, and negative attitudes specifically against Muslims are a real and increasing challenge both in Norway and other parts of the world. We therefore took religion as a proxy to define word lists for this ethnicity, comparing Christian religion and Islam in tests NO2 and NO3. Again, positive and negative sentiment was assessed in NO3 (as in the original test in Caliskan et al. (2017)), and potential differences between the concepts of success and failure in NO2 (following Kurpicz-Briki and Leoni, 2021).

The topic of candidates having children or potentially having children (in the case of a female candidate: “she is young and married, will she have children soon?” (*hun er ung og gift, vil hun få barn snart?*). We therefore created two tests, putting words concerning career and having children (extending the concept in the original WEAT (Caliskan et al., 2017) of family to a more specific dimension) in relation to women and men (NO5) and people that are single vs. people that are in a couple (NO6). (Ely et al., 2014; Offermann et al., 2020; Padavic et al., 2020) argue for moving beyond the narrative of women workers as *mothers with no time* to be aware of different types of female employees. Single young women have as a worker category been described as fitting neither masculine nor feminine leadership expectations by Merluzzi and Phillips (2022). In their study of *Marital status bias in perceptions of employees* (Jordan and Zitek, 2012) finds that “Female employees or potential employees may be viewed as less suitable for employment when married than when single, whereas the reverse may be true for men”.

Taken together, the Norwegian co-creation workshops resulted in seven bias test categories that reflect key stereotypes discussed by participants. *NO1* captures sentiment associations between Iranian and Norwegian names (negative vs. positive), while *NO2* and *NO3* assess religious bias by comparing Muslim and Christian identities in relation to success, failure, and general sentiment. *NO4* revisits ethnic bias, linking Iranian and Norwegian names to notions of competence and achievement (failure vs. success). *NO5* examines gendered expectations by contrasting male and female identities with career versus family orientation, and *NO6* extends this comparison to relationship status (single vs. in a couple). Finally, *NO7* targets the stereotype that “Iranians are good at selling,” contrasting Iranian and Norwegian names with associations of sales ability. Together, these tests form a comprehensive and culturally grounded framework for analyzing social bias in Norwegian language models.

#### 2.4.4 Icelandic

Based on the profiles of the Icelandic co-creation workshop, different discussions around Asians and Icelandic individuals were documented in the report and the word lists from the workshop. In particular, the stereotype of the “Asian technology nerd” (*As*í*skur tækninörd*) was mentioned. Additionally, a discussion around whether “somebody Asian can be the face of the [Icelandic] company” (*As*í*skt andlit sem andlit fyrirtækis*) took place. Based on this, different WEAT tests were designed. IS1 investigates the general positive and negative sentiment of Icelandic and Chinese surnames, inspired by the original WEAT (Caliskan et al., 2017) from the US-American context (considering African-American and European-American names). IS2 uses the same surnames, however, with a focus on the technology stereotype. Similarly, IS5 investigates wording related to success and failure for the two groups, inspired by the existing test in Kurpicz-Briki and Leoni (2021). IS3 and IS6 are variants of some of the previously mentioned tests, by using European and Asian country names rather than individual's surnames.

Although Iceland has ranked first on the Global Gender Gap Index since 2009, which evaluates gender equality in 146 countries,^15^ gender biases are as of today still a relevant topic for the Icelandic society, e.g., concerning disparities in top-level positions in academia (Carlsson et al., 2021; Hjálmsdóttir and Rafnsdóttir, 2022) and in business leadership (Axelsdóttir et al., 2023), or the media (Jóhannsdóttir and Einarsdóttir, 2015).

For one profile in the co-creation workshop, participants discussed that being pretty in physical appearance could impact their success both in a positive or negative way. This is in line with research investigating the relationship between body weight and employment in Iceland (Asgeirsdottir, 2011; Rúdólfsdóttir and Jóhannsdóttir, 2018). We cover this investigation by the newly created test IS4.

One candidate was named as a “power-lesbian” (*powerlessa*) during the workshop, which brought up the discussion whether the stereotype of being gay or lesbian is necessarily negative or whether there can be positive associations. Different combinations are tested in IS7, IS7 v2, IS8 and IS8 v2.

In general, the Icelandic co-creation workshop led to the development of ten new bias test categories that address stereotypes related to ethnicity, technology, gender, beauty, and sexual orientation. *IS1* measures sentiment differences between Icelandic and Chinese surnames (positive vs. negative), while *IS2* examines associations between these groups and domains such as leisure versus technology, reflecting the “Asian technology nerd” stereotype. *IS3* mirrors this setup using European and Asian country names instead of surnames, and *IS5* evaluates the same name sets with a focus on success and failure, followed by *IS6*, which applies the success-failure dimension to the country name variant. *IS4* investigates perceptions of physical appearance and career success, capturing beauty-related bias. Finally, *IS7* and its variant *IS7 v2* assess the bias toward sexual orientation and gender identity (gay/lesbian or non-binary/trans vs. straight) in relation to success and failure, while *IS8* and *IS8 v2* test the polarity of the feelings (negative vs. positive) for the same groups. Collectively, these tests reflect the social themes discussed in the workshops and provide a culturally grounded framework for assessing representational bias in Icelandic language models.

#### 2.4.5 Turkish

The outcomes of the co-creation workshop indicated that candidate's attributes that were considered positive were work and personality-related, e.g., hardworking, smart, good English skills, detailed and meticulous work profile, communication skills. An exception to these positive attributes, however, was noted in the form of excessive confidence (“high ego”). On the other side, aspects unrelated to work were identified as risks of bias by the participants of the co-creation workshop regardless of gender of the mock applicant. These include family attributes (marital status, fact of having children, being newly married, etc.) as well as personal life attributes (e.g., engaging in multiple hobbies, military obligations, an unprofessional appearance in photographs, wealth, confidence, desire to travel). Therefore, we investigate the stereotype of being less productive when having a family (TR1), or hobbies (TR2). We also consider the general male/female stereotype with positive and negative expressions from the co-creation workshops (TR3), as literature has pointed out the discrimination of women on the Turkish labor market, e.g., Akgul (2018).

Together, the Turkish co-creation workshop led to the development of three categories of bias tests that reflect stereotypes related to productivity, family, leisure, and gender. *TR1* contrasts career-oriented and family-oriented concepts (productive vs. unproductive, career vs. family), capturing the stereotype that having a family reduces productivity or professional commitment. *TR2* follows a similar structure but replaces family with hobbies (productive vs. unproductive, career vs. hobbies), examining perceptions of dedication and work ethic. Finally, *TR3* explores gender-related associations by contrasting male and female identities through positive and negative expressions or interests, reflecting the persistent gender bias reported in the Turkish labor market. Together, these categories provide a culturally grounded framework for evaluating bias in Turkish language models.

## 3 Results

### 3.1 Measuring bias with the BIAS-WEAT and BIAS-SEAT metrics

This section shows the results of the different bias detection metrics that were implemented and described previously. For each language, we first present the results for BIAS-WEAT on static word embeddings (*fasttext*). We then present the results for the BIAS-SEAT metric on contextualized word embeddings (*BERT*-based models). For each language, different experiments have been conducted: specific tests derived from the co-creation activities (e.g., IS1-IS8 for Icelandic), and translated tests from other sources (e.g., the GER and WEAT tests mentioned in the results table for comparison).

The translated tests serve as a comparison with other comparable work in the field. Details about GER1 and GER2 can be found in Kurpicz-Briki (2020), details about the original WEAT tests in Caliskan et al. (2017). The results presented in this paper have been based on translated tests from the interdisciplinary BIAS consortium. In some cases, translation was possible in different variants or there were discussions among the consortium. In these cases, different variants have been tested, e.g., WEAT6_v1 and WEAT6_v2 for German. The datasets with the entire word lists for the tests are shared in our Github repository.^16^ The tests translated in the consortium were translated manually by native speakers of the corresponding language in a shared spreadsheet, discussing disagreement and alternatives within the team.

In the following, there will be a subsection for each language describing the models and the results. The results are presented in a table with one line for each test. A test corresponds to a specific concept, the exact tests for each language have been described in Section 2.4.

It also has to be pointed out that even when results do not demonstrate a statistically significant bias (a statistically significant bias is marked with a ✓in the table), it cannot be concluded that there is no bias with regard to the concerned test. Following Caliskan et al. (2017), statistical significance was assessed using one-sided permutation-based *p*-values, where a measured effect size is considered significant if *p* < 0.05. The use of one-sided *p*-values implies the expectation that any detected bias will be *stereotypical*. Interestingly, several of the tests conducted in this work detected *anti-stereotypical* bias, characterized by a negative effect size and *p*>0.95. Consistent with common practice in WEAT implementations and related work, 10,000 permutation iterations were used for approximation.

#### 3.1.1 German

##### 3.1.1.1 Models

The BIAS-WEAT tests are applied to static word embeddings. The corresponding German fasttext embeddings^17^ were used. To calculate the p-value, 10K iterations were used. For assessing bias in contextualized word embeddings using the BIAS-SEAT metric, sentence templates rather than word lists were used. Two different contextualized BERT-based models (google-bert/bert-base-german-cased^18^ and dbmdz/bert-base-german-cased^19^) have been tested. To calculate the p-value, 10K iterations were used.

##### 3.1.1.2 BIAS-WEAT

Table 1 gives an overview of the results, specifying the effect size of the bias, and whether the observed bias was statistically significant (*p* < 0.05). In addition to our newly created tests (CW1-CW5), the results for the translated tests [GER1/2 as in Kurpicz-Briki (2020) and WEAT tests from English (Caliskan et al., 2017)] are displayed.

**Table 1:** Results of the experiments testing the German fasttext embeddings.

Name	*p*-value	Effect size	Bias Detected?
CW1 (family/productivity)	0.0017	1.256029	✓
CW2 (prodictivity/age)	0.0219	0.9085668	✓
CW3 (social belonging/cultural identity)	0.1394	0.6705451	✗
CW4 (male/female hobbies)	0.0001	1.4601815	✓
CW5 (gender/productivity)	0.0033	1.1028917	✓
GER1 (gender/studies)	0.0001	1.8303912	✓
GER2 (gender/character)	0.002	1.4895244	✓
WEAT_5 (origin/pos&neg)	0.0005	1.1521959	✓
WEAT_6_v1 (gender/career)	0.0001	1.674007	✓
WEAT_6_v2 (gender/career)	0.0014	1.486258	✓
WEAT_7 (gender/math&art)	0.6774	-0.24060686	✗
WEAT_8 (gender/science&art)	0.4166	0.118204206	✗

✓means a bias was detected (statistically significant).

##### 3.1.1.3 BIAS-SEAT

Tables 2, 3 gives an overview of the results, specifying the effect size of the bias, and whether the observed bias was statistically significant (*p* < 0.05).

**Table 2:** Results testing the model: google-bert/bert-base-german-cased.

Name	*p*-value	Effect Size	Bias detected?
CW1 (family/productivity)	0.0001	1.0469131	✓
CW2 (prodictivity/age)	0.0001	0.99556607	✓
CW3 (social belonging/cultural identity)	0.7432	-0.13594475	✗
CW4 (male/female hobbies)	0.0001	1.1129073	✓
CW5 (gender/productivity)	0.0003	0.5501282	✓
GER1 (gender/studies)	0.0043	0.58287966	✓
GER2 (character/gender)	0.0001	0.988066	✓

✓means a bias was detected (statistically significant).

**Table 3:** Results testing the model: dbmdz/bert-base-german-cased.

Name	*p*-value	Effect size	Bias detected?
CW1 (family/productivity)	0.0001	0.9029751	✓
CW2 (prodictivity/age)	0.0026	0.43382937	✓
CW3 (social belonging/cultural identity)	0.4491	0.029363483	✗
CW4 (male/female hobbies)	0.0015	0.65302795	✓
CW5 (gender/productivity)	0.0001	0.94554406	✓
GER1 (gender/studies)	0.0016	0.63156605	✓
GER2 (character/gender)	0.0001	1.3069388	✓

✓means a bias was detected (statistically significant).

##### 3.1.1.4 Discussion

We observe that some biases can clearly be demonstrated over the different models. For example, GER1 (gender and study subjects) and GER2 (historical gender biases), originally developed in Kurpicz-Briki (2020), have a significant bias in all the models. This is also the case for our new tests CW1 (productivity vs career/family), CW2 (productivity vs age), CW4 (male/female and hobbies) and CW5 (productivity vs gender). On the other side, no statistically significant bias was identified in the CW3 (traditionalists/communicator vs native/immigrant). We assume that the reason could potentially be the setup of the test, because *traditionalists* might not be the direct counterparts of *communicators*, as assumed for simplicity in the test. A a more detailed discussion is provided in our paper (Rankwiler and Kurpicz-Briki, 2024).

#### 3.1.2 Dutch

##### 3.1.2.1 Models

The BIAS-WEAT tests are applied to static word embeddings. The corresponding Dutch fasttext embeddings^20^ were used. To calculate the *p*-value, 10K iterations were used. For assessing bias in contextualized word embeddings using our metric BIAS-SEAT, sentence templates rather than word lists are used. Two different contextualized BERT-based models have been used - GroNLP/bert-base-dutch-cased (de Vries et al., 2019) and pdelobelle/robbert-v2-dutch-base (Delobelle et al., 2020). To calculate the p-value, 10K iterations were used.

##### 3.1.2.2 BIAS-WEAT and BIAS-SEAT

Table 4 gives an overview of the results, specifying the effect size of the bias, and whether the observed bias was statistically significant (p < 0.05). In addition to our newly created tests (CW1, CW2), the results for the translated tests [GER1/2 from German (Kurpicz-Briki, 2020) and WEAT tests from English (Caliskan et al., 2017)] are displayed.

**Table 4:** Combined results of BIAS-WEAT and BIAS-SEAT bias evaluations for Dutch.

Test name	Model/embedding	*p*-value	Effect size	Bias detected?
CW1_NL (disability/productivity)	fastText	0.3068	0.3133	✗
CW2_NL (LGBTQIA+/psychological stabiliy)	fastText	0.7649	-0.4669	✗
GER1_NL (gender/studies)	fastText	0.0155	1.4196	✓
GER2_NL (character/gender)	fastText	0.0039	1.4828	✓
WEAT7_MT_NL (gender/math&art)	fastText	0.0071	1.1972	✓
WEAT8_MT_NL (gender/science&art)	fastText	0.1188	0.6422	✗
SEAT_CW1_NL (disability/productivity)	BERTje	0.0004	0.5455	✓
**SEAT_CW2_NL (LGBTQIA+/psychological stabiliy)**	BERTje	0.9997	-0.5836	✓
SEAT_GER1_NL (gender/studies)	BERTje	0.0049	0.5635	✓
SEAT_GER2_NL (character/gender)	BERTje	0.4683	0.0182	✗
SEAT_CW1_NL (disability/productivity)	RobBERT	0.6085	-0.0434	✗
SEAT_CW2_NL (LGBTQIA+/psychological stabiliy)	RobBERT	0.6085	-0.0447	✗
SEAT_GER1_NL (gender/studies)	RobBERT	0.8385	-0.2244	✗
SEAT_GER2_NL (character/gender)	RobBERT	0.0060	0.5075	✓

fastText refers to word embeddings, while BERTje and RobBERT are Dutch sentence encoders. Bias is considered statistically significant when p < 0.05 (✓means a bias was detected (statistically significant)). Boldfaced test names indicated instances where the detected bias was *antisterotypical*.

##### 3.1.2.3 Discussion

Even though the word lists CW1 (disability and productivity) and CW2 (regarding sexual orientation/identify and psychological stability) were derived from the topics of the co-creation activities, a statistically significant bias could only be identified in the BERTje model. Interestingly, the two wordlists translated from German (GER1 and GER2), showed statistically significant biases in some models, but not in others. Also of interest is that CW2 detected significant anti-sterotypical bias for BERTje. Versions of the translations of WEAT6, WEAT7 and WEAT8 can also be found in the literature (Chávez Mulsa and Spanakis, 2020).

#### 3.1.3 Norwegian

##### 3.1.3.1 Models

The BIAS-WEAT tests are applied to static word embeddings. The corresponding Norwegian fasttext embeddings^21^ were used. To calculate the p-value, 10K iterations were used. For assessing bias in contextualized word embeddings using our BIAS-SEAT metric, sentence templates rather than word lists are used. Three different contextualized BERT-based models have been used: ltg/norbert3-base (Samuel et al., 2023), NbAiLab/nb-bert-base^22^ and Twitter/twhin-bert-base (Zhang et al., 2022). To calculate the *p*-value, 10K iterations were used.

##### 3.1.3.2 BIAS-WEAT

Table 5 gives an overview of the results, specifying the effect size of the bias, and whether the observed bias was statistically significant (*p* < 0.05). In addition to our newly created tests (NO1-NO7), the results for the translated tests [GER1/2 from German (Kurpicz-Briki, 2020) and WEAT tests from English (Caliskan et al., 2017)] are displayed.

**Table 5:** Results for testing bias in Norwegian fasttext word embeddings.

Name	*p*-value	Effect size	Bias detected?
WEAT_8 (gender/math&science)	0.1112	0.6550296	✗
WEAT_7 (gender/math&art)	0.0551	0.821902	✗
WEAT_6 (gender/career)	0.0033	1.1463478	✓
GER1 (gender/studies)	0.0001	1.45817	✓
GER2 (character/gender)	0.1523	0.6751104	✗
NO1 (origin/pos&neg)	0.2292	0.35538986	✗
NO1_v2 (origin/pos&neg)	0.0002	1.5705495	✓
NO2 (religion/success)	0.0438	0.9485602	✓
NO3 (religion/pos&neg)	0.012	1.2478302	✓
NO3_v2 (religion/pos&neg)	0.0146	1.1976784	✓
NO4 (origin/success)	0.111	0.5820447	✗
NO5 (gender/career)	0.0003	1.4556111	✓
NO6 (family/career)	0.0155	1.2967607	✓
NO7 (origin/sales ability)	0.0004	1.2549357	✓

✓means a bias was detected (statistically significant).

##### 3.1.3.3 BIAS-SEAT

Tables 6–8 give an overview of the results, specifying the effect size of the bias, and whether the observed bias was statistically significant (*p* < 0.05).

**Table 6:** Testing bias with SEAT-BIAS in the BERT-based Norwegian model ltg/norbert3-base.

Name	*p*-value	Effect size	Bias detected?
SEAT_WEAT_8 (gender/math&science)	0.5521	-0.021236954	✗
**SEAT_WEAT_7 (gender/math&art)**	0.9869	-0.36104167	✓
SEAT_WEAT_6 (gender/career)	0.09	0.2457135	✗
SEAT_GER1 (gender/studies)	0.0002	0.8200612	✓
SEAT_GER2 (character/gender)	0.0578	0.3990067	✗
SEAT_NO1 (origin/pos&neg)	0.6616	-0.077566765	✗
SEAT_NO1_v2 (origin/pos&neg)	0.1412	0.19629392	✗
SEAT_NO2 (religion/success)	0.3342	0.08737803	✗
SEAT_NO3 (religion/pos&neg)	0.0024	0.588982	✓
SEAT_NO3_v2 (religion/pos&neg)	0.9034	-0.2719466	✗
SEAT_NO4 (origin/success)	0.1285	0.20591259	✗
SEAT_NO5 (gender/career)	0.1659	0.17252547	✗
SEAT_NO6 (family/career)	0.0332	0.6705886	✓
SEAT_NO7 (origin/sales ability)	0.2976	0.09768746	✗

✓means a bias was detected (statistically significant). Boldfaced test names indicated instances where the detected bias was *antisterotypical*.

**Table 7:** Testing bias with BIAS-SEAT in the model NbAiLab/nb-bert-base.

Name	*p*-value	Effect size	Bias detected?
SEAT_WEAT_8 (gender/math&science)	0.0001	0.94059145	✓
SEAT_WEAT_7 (gender/math&art)	0.0001	0.69606787	✓
SEAT_WEAT_6 (gender/career)	0.0146	0.40809816	✓
SEAT_GER1 (gender/studies)	0.0018	0.72742033	✓
SEAT_GER2 (character/gender)	0.0001	1.153834	✓
SEAT_NO1 (origin/pos&neg)	0.0001	1.1169631	✓
SEAT_NO1_v2 (origin/pos&neg)	0.0317	0.33976817	✓
SEAT_NO2 (religion/success)	0.6599	-0.08469217	✗
SEAT_NO3 (religion/pos&neg)	0.0001	1.345268	✓
SEAT_NO3_v2 (religion/pos&neg)	0.0001	1.132821	✓
**SEAT_NO4 (origin/success)**	0.9999	-0.677313	✓
SEAT_NO5 (gender/career)	0.0001	0.78993636	✓
SEAT_NO6 (family/career)	0.0004	1.1687391	✓
SEAT_NO7 (origin/sales ability)	0.3953	0.04946232	✗

✓means a bias was detected (statistically significant). Boldfaced test names indicated instances where the detected bias was *antisterotypical*.

**Table 8:** Bias detection results with BIAS-SEAT in the model Twitter/twhin-bert-base.

Name	*p*-value	Effect size	Bias detected?
SEAT_WEAT_8 (gender/math&science)	0.1755	0.15662152	✗
SEAT_WEAT_7 (gender/math&art)	0.8712	-0.18175985	✗
**SEAT_WEAT_6 (gender/career)**	0.9802	-0.3765248	✓
SEAT_GER1 (gender/studies)	0.2034	0.20712435	✗
SEAT_GER2 (character/gender)	0.0034	0.65253276	✓
**SEAT_NO1 (origin/pos&neg)**	0.9904	-0.43127236	✓
SEAT_NO1_v2 (origin/pos&neg)	0.6863	-0.08627387	✗
SEAT_NO2 (religion/success)	0.5948	-0.047226407	✗
SEAT_NO3 (religion/pos&neg)	0.9412	-0.32603282	✗
SEAT_NO3_v2 (religion/pos&neg)	0.7458	-0.1315192	✗
SEAT_NO4 (origin/success)	0.7942	-0.1470713	✗
**SEAT_NO5 (gender/career)**	0.9966	-0.49224302	✓
SEAT_NO6 (family/career)	0.0042	0.96374273	✓
SEAT_NO7 (origin/sales ability)	1.0001	-0.6706777	✗

✓means a bias was detected (statistically significant). Boldfaced test names indicated instances where the detected bias was *antisterotypical*.

##### 3.1.3.4 Discussion

For Norwegian, different models exposed different biases. Whereas a significant bias was identified for example for GER1 (gender and study subjects) in three of the models, and not for GER2 (historical gender biases), the results indicate the opposite for the model Twitter/twhin-bert-base (Zhang et al., 2022). In general, less significant biases were identified with this model. This is interesting, because it is based on social media data. For the same model, several tests even detected anti-stereotypical bias, albeit with relatively small effect sizes. The test NO6 about being in a couple/single and career/having children led to a statistically significant bias in all the tested models.

#### 3.1.4 Icelandic

##### 3.1.4.1 Models

The BIAS-WEAT tests are applied to static word embeddings. The corresponding Icelandic fasttext embeddings^23^ were used. To calculate the *p*-value, 10K iterations were used. For assessing bias in contextualized word embeddings using our BIAS-SEAT metric, sentence templates rather than word lists are used. The Icelandic BERT-based model mideind/IceBERT (Snæbjarnarson et al., 2022) was tested. To calculate the *p*-value, 10K iterations were used.

##### 3.1.4.2 BIAS-WEAT

Table 9 gives an overview of the results, specifying the effect size of the bias, and whether the observed bias was statistically significant (*p* < 0.05). In addition to our newly created tests (IS1-IS8), the results for the translated tests [GER1/2 from German (Kurpicz-Briki, 2020) and WEAT tests from English (Caliskan et al., 2017)] are displayed.

**Table 9:** Testing bias with BIAS-WEAT for the *fasttext* word embeddings in Icelandic.

Name	*p*-value	Effect size	Bias detected?
GER1 (gender/studies)	0.035	1.3354226	✓
GER1_v2 (gender/studies)	0.0622	1.0069298	✗
GER2 (character/gender)	0.4866	0.025816023	✗
GER2_v2 (character/gender)	0.1166	0.7278196	✗
IS1 (origin/pos&neg)	0.0001	1.5525488	✓
IS2 (origin/technology - names)	0.003	1.1665666	✓
IS3 (origin/technology - countries)	0.7468	-0.315216	✗
IS4 (physical appearance/success)	0.0003	1.4641095	✓
IS5 (origin/success - names)	0.5774	-0.09725275	✗
IS6 (origin/success - countries)	0.6032	-0.12510304	✗
IS6_v2 (origin/success - countries)	0.0085	1.0376033	✓
IS7 (sex. orientation, gender identity/success)	0.8341	-1.3575592	✗
IS7_v2 (sex. orientation, gender identity/success)	0.8354	-1.337458	✗
IS8 (sex. orientation, gender identity/pos&neg)	0.503	-0.32880002	✗
IS8_v2 (sex. orientation, gender identity/pos&neg)	0.6701	-1.2624458	✗
WEAT_6 (gender/career)	0.0188	0.9594196	✓
WEAT_7 (gender/math&art)	0.4793	0.01702043	✗
WEAT_7_v2 (gender/math&art)	0.0431	0.8988872	✓
WEAT_7_v3 (gender/math&art)	0.0444	0.88183177	✓
WEAT_8 (gender/science&art)	0.5179	-0.022100868	✗
WEAT_8_v2 (gender/science&art)	0.4554	0.060245737	✗

✓means a bias was detected (statistically significant).

##### 3.1.4.3 BIAS-SEAT

Table 10 gives an overview of the results, specifying the effect size of the bias, and whether the observed bias was statistically significant (*p* < 0.05).

**Table 10:** Testing bias with BIAS-SEAT in the model mideind/IceBERT.

Name	*p*-value	Effect size	Bias detected?
SEAT_WEAT_8 (gender/science&art)	0.3669	0.13278891	✗
SEAT_WEAT_7_v3 (gender/math&art)	0.1553	0.3857893	✗
SEAT_WEAT_6 (gender/career)	0.4543	0.0385408	✗
SEAT_GER1 (gender/studies)	0.4211	0.093760006	✗
SEAT_IS1 (origin/pos&neg)	0.4565	0.041696977	✗
SEAT_IS2 (origin/technology - names)	0.2932	0.17944542	✗
SEAT_IS3 (origin/ technology - countries)	0.0043	0.82488626	✓
SEAT_IS4 (physical appearance/success)	0.0001	1.1541324	✓
SEAT_IS5 (origin/success - names)	0.2335	0.24088281	✗
SEAT_IS6 (origin/success - countries)	0.9419	-0.49553496	✗
SEAT_IS6_v2 (origin/ success - countries)	0.1039	0.40585342	✗
SEAT_IS7 (sex. orientation, gender identity/success)	0.7112	-0.3796883	✗
SEAT_IS7_v2 (sex. orientation, gender identity/success)	0.6615	-0.3398584	✗
SEAT_IS8 (sex. orientation, gender identity/pos&neg)	0.3282	0.35750616	✗
SEAT_IS8_v2 (sex. orientation, gender identity/pos&neg)	0.0153	1.0745541	✓

✓means a bias was detected (statistically significant).

##### 3.1.4.4 Discussion

As for Norwegian, different models lead to different statistically significant biases. For example, the bias tests for IS1 and IS2 (Icelandic and Chinese surnames vs. pleasant/unpleasant and technology words respectively) were positive in the fasttext word embedding, however, both were not statistically significant in the IceBERT model. IS4 investigating the relation between beauty and success, was significant in both models. In the translated tests (e.g., GER1, GER2) some challenges were encountered that might have impacted the results, such as the number of different morphological forms in Icelandic, or the difficulty of translating existing texts without having out-of-vocabulary words, especially with the fasttext embeddings.

#### 3.1.5 Turkish

##### 3.1.5.1 Models

The BIAS-WEAT tests are applied to static word embeddings. The corresponding Turkish fasttext embeddings,^24^ were used. To calculate the *p*-value, 10K iterations were used. For assessing bias in contextualized word embeddings using our BIAS-SEAT metric, sentence templates rather than word lists are used. The Turkish BERT model dbmdz/bert-base-turkish-cased^25^ was tested. To calculate the p-value, 10K iterations were used.

##### 3.1.5.2 BIAS-WEAT

Table 11 gives an overview of the results, specifying the effect size of the bias, and whether the observed bias was statistically significant (*p* < 0.05). In addition to our newly created tests (IS1-IS8), the results for the translated tests (GER1/2 from German (Kurpicz-Briki, 2020) and WEAT tests from English (Caliskan et al., 2017)) are displayed.

**Table 11:** Testing bias with BIAS-WEAT for the Turkish *fasttext* model.

Name	*p*-value	Effect size	Bias detected?
GER1 (gender/studies)	0.0007	1.5902035	✓
GER2 (character/gender)	0.2146	0.44277787	✗
TR1 (productivity/family)	0.0054	1.1070601	✓
TR2 (productivity/hobbies)	0.1369	0.5171289	✗
TR3 (gender/pos&neg)	0.0002	1.0562279	✓
WEAT_6 (gender/career)	0.0001	1.5016291	✓
WEAT_7 (gender/math&art)	0.003	1.3136436	✓
WEAT_8 (gender/science&art)	0.5069	-0.0016555324	✗

✓means a bias was detected (statistically significant).

##### 3.1.5.3 BIAS-SEAT

Table 12 gives an overview of the results, specifying the effect size of the bias, and whether the observed bias was statistically significant (*p* < 0.05).

**Table 12:** Testing bias with BIAS-SEAT in the model dbmdz/bert-base-turkish-cased.

Name	*p*-value	Effect size	Bias detected?
SEAT_TR1 (productivity/family)	0.5803	-0.054122563	✗
SEAT_TR2 (productivity/hobbies)	0.5818	-0.06297266	✗
SEAT_TR3 (gender/pos&neg)	0.0172	0.3281853	✓

✓means a bias was detected (statistically significant).

##### 3.1.5.4 Discussion

The test TR3 concerning gender bias (male/female names vs. interests that are considered positive or negative for recruiting in the co-creation workshop) had a statistically significant bias in both models. For the other tests, as in other languages, different biases were significant in different models. TR2 dealing with productivity and hobbies did not show a significant bias in either of the models. This is interesting as it was explicitly mentioned in the co-creation activities.

### 3.2 Cross-linguistic and model-dependent patterns in bias detection

Across languages and models, the results reveal substantial variation in both the presence and strength of detected biases. While some bias dimensions, such as associations between productivity and family status or gender, are consistently reflected across multiple languages and models, other biases identified during the co-creation workshops yield weaker, inconsistent, or statistically non-significant results. This variability indicates that representational bias in language models is not uniformly expressed across linguistic and sociocultural contexts. Instead, it reflects context-specific social dynamics and differences in training data composition and model architecture.

A comparison between co-created bias tests and translated benchmark tests further highlights some methodological differences. Translated tests derived from established WEAT or GER benchmarks frequently exhibit statistically significant biases across languages, particularly for well-established dimensions such as gender-career or sentiment-based associations. In contrast, several co-created tests show mixed or model-dependent outcomes. Rather than indicating a limitation of the co-created approach, these results point to a distinction between detecting broadly entrenched stereotypes and identifying more localized or context-sensitive forms of bias. Co-created tests are designed to capture socially salient biases grounded in specific institutional and cultural settings, which may not always be strongly encoded in general-purpose language models or may be expressed in more subtle ways.

Finally, the results emphasize the sensitivity of bias detection outcomes to the choice of language model and training data. Differences observed between static word embeddings and contextualized models, as well as among contextualized models trained on different corpora (e.g., web text versus social media), indicate that representational bias is closely tied to the linguistic environments reflected in the underlying data. In several cases, the same bias test yields statistically significant results in one model but not in another, highlighting the importance of considering model provenance and domain when interpreting bias detection results.

## 4 Discussion

### 4.1 Sociotechnical contextualization

This study arises from a significant gap in the state of the art: machine learning models trained on biased data not only risk reproducing and amplifying existing social inequalities but also tend to privilege Western, English-speaking contexts while demonstrating reduced reliability in low-resource languages. Such English-centric orientations constrain global applicability, reinforce stereotypes derived from unrepresentative training sources, and hinder the evaluation of bias in complex decision-making domains such as selection and recruitment.

In response, this study applied bias detection methods beyond English to contribute to a more comprehensive understanding of how linguistic and cultural diversity shape the reliability and fairness of machine learning models. Through co-creation workshops with native speakers, it confirmed that biases linked to personal characteristics, such as gender and migration status in Germany, or disability and LGBTIQA+ identity in the Netherlands, as well as perceptions of work-related traits such as productivity, are context-dependent and culturally embedded. The empirical results presented in Section 3 further demonstrate that these contextually grounded bias dimensions are not uniformly reflected across languages or models, but instead exhibit substantial variability in their presence and statistical strength. These findings resonate with broader interdisciplinary literature emphasizing that bias is not a universal or technical flaw but a sociotechnical phenomenon reflecting local norms, power relations, and historical inequalities (Fundamental Rights Agency, 2022; O'Neil, 2016; Selbst and Vertesi, 2019).

The co-creation workshops operationalize the project's commitment to embedding contextual expertise directly into the design of the bias-metric pipeline (Sections 2 and 3). By eliciting stakeholder-generated linguistic data, grounded in real recruitment practices and shaped by sectoral patterns of gendered and racialized segregation, the workshops provide a situated complement to the computational models developed in subsequent work within the project. This process ensures that the lexical resources required for WEAT-based assessments (Section 3) are not abstractly defined but instead reflect empirically observed associations and domain-specific forms of bias. As evidenced by the mixed and model-dependent outcomes of several co-created tests, this approach prioritizes social relevance and contextual legitimacy over the assumption that all salient biases will be strongly encoded in general-purpose language models. The co-creation component therefore acts as a sociotechnical interface: it translates experiential, normative and professional knowledge into structured lexical inputs, enabling the bias metric to remain sensitive to context, to intersectional dynamics, and to the practical constraints of HR decision-making environments.

Our findings also complement other results produced within the BIAS project. Its large-scale survey^26^ of 4,317 respondents across the European Union, Iceland, Norway, Switzerland, and Türkiye, together with 71 expert interviews^27^ with HR practitioners and AI developers in Estonia, Iceland, Italy, the Netherlands, Norway, Switzerland, France and Türkiye, offered significant insights into how various stakeholders perceive AI in the labor market. While both respondents and experts generally considered the use of AI in recruitment fairly positively, concerns about diversity bias persisted, particularly among groups socially marginalized and historically oppressed in the labor market. The alignment between these perceptions and the bias dimensions, as accentuated through co-creation, underlines the value of grounding technical evaluation in stakeholder-informed understandings of harm.

From a Social Sciences and Humanities (SSH) perspective, this study's contribution to culturally grounded and socially responsive methods for bias detection and mitigation aligns with the European broader effort to promote trustworthy technological progress.^28^ More broadly, it illustrates how interdisciplinary approaches that integrate technical reliability with social understanding are essential to advancing fairness and accountability in AI systems that are used in the labor market across linguistic and cultural boundaries (Rigotti and Fosch-Villaronga, 2024). The variability and context-dependence observed in the results caution against universal, context-blind interpretations of bias metrics, reinforcing the need for sociotechnical evaluation frameworks.^29^

The findings of this study align with qualitative studies conducted in the BIAS-project on discrimination,^30^ where we explored how AI is used in multiple parts of the HR process. This included a) assessing candidates with and without the help of AI, b) managing workers through a quantification of their performance, c) practical and imagined impact of AI on the effectiveness and fairness of HR management systems, as well as d) counter strategies from the job-applicants sides when faced with AI systems (applying AI systems of their own to the recruitment process). Taken together, these converging strands of evidence situate the present results within a broader sociotechnical landscape in which bias detection must be understood as both a technical and a socially embedded practice.

### 4.2 Limitations

One challenge identified throughout this work was encountered with languages with less resources in the field of natural language processing research available. Words or concepts mentioned in the workshops were not always included in some of the corresponding language models' vocabulary. For example, due to the limited available training data for some languages in the time the models were trained, or the limitations of some types of models to deal with out-of-vocabulary words. In those cases, other suitable or similar wording was identified. These constraints are not merely technical but reflect broader inequalities in language representation within existing NLP resources, which may influence both the detectability and the strength of observed bias effects across languages.

Regarding the overall method, it is probable that our work is to some extent subject to a selection bias, as we picked up topics from what was mentioned in the co-creation activities executed in the project. Not all mentioned topics were covered, and, additionally, the mentioned topics were highly dependent on the proposed profiles that were discussed in the co-creation workshops. This dependency on situated workshop dynamics is an inherent characteristic of participatory methods, as they surface biases that are salient within specific institutional and sociocultural contexts rather than aiming for exhaustive or universal coverage. However, our method is defined in a generic way to easily include additional forms of bias in the future and extend our project that will be released as open source. In this sense, the framework should be understood as extensible rather than complete.

It has to be noted that, even in cases where no significant bias is identified by our methods, it cannot be implied that there is no bias. Other blind spots of our method and thus undiscovered biases might be contained in the models that are not covered with the selection of bias tests based on the co-creation activities. As demonstrated in Section 3, the absence or inconsistency of statistically significant results may reflect weak or indirect encoding of certain biases in the underlying models, competing associations, or limitations of current evaluation paradigms, rather than the absence of socially relevant bias. This is general limitation of the state-of-the-art methods based on WEAT/SEAT. Accordingly, bias detection results should be interpreted as indicative rather than definitive, and always in relation to the sociotechnical context in which both the models and the evaluation methods are situated.

Another limitation of this work is that the experiments rely on traditional word embedding approaches, which were selected due to the original multilingual study design and the need to model each language separately. While these methods remain suitable for analyzing multilingual representations in our setting, recent advances in large language models (LLMs) may enable richer contextual representations and potentially improve downstream performance. The proposed methodology is not restricted to static embeddings and can be extended to LLM-based representations; to facilitate such extensions, the implementation is publicly available on GitHub^31^. Exploring and systematically evaluating LLM-based variants constitutes an important direction for future work, particularly given the rapid pace of developments in natural language processing technologies.

## Data Availability

The datasets presented in this study can be found in online repositories. The names of the repository/repositories and accession number(s) can be found in the article/supplementary material.

## Footnotes

2
https://www.biasproject.eu

3
https://github.com/BFH-AMI/BIAS

4 Deliverable available at https://www.biasproject.eu/public_report/final-report-on-co-creation-methodologies-and-findings/.

5
https://github.com/BFH-AMI/BIAS-DetectionFramework

6 The results for Italian will not be presented in this paper explicitly, as they have been published in Puttick and Kurpicz-Briki (2025).

7 Different word types have different templates, so, for example, verbs have fewer sentences than nouns. Depending on the word type composition of a word list, the sentence list will have a different number of sentences. To conduct the test, targets and attributes must have the same number of sentences respectively. In some cases, the overall number of sentences was limited to ensure computational efficiency of the test.

8
https://spacy.io/

9 The results shown in this subsection for German were previously already published at a conference (Rankwiler and Kurpicz-Briki, 2024).

10
https://www.equalvoice.ch/wp-content/uploads/2024/07/BCG-Whitepaper-what-keeps-women-out-of-labor-force-switzerland-1.pdf

11
https://www.bfs.admin.ch/bfs/en/home/statistics/population/migration-integration/integration.html

12
https://www.transgendernetwerk.nl/wp-content/uploads/2023/01/Inclusion4All-National-report-Netherlands_EN-Transgender-Netwerk.pdf

13 Data Source: https://www.ssb.no/en/befolkning/navn/statistikk/navn, names from 1990, accessed 11/2023.

14 Data Source: https://studentsoftheworld.info/penpals/stats.php?Pays=IRN, accessed 11/2023.

15
https://www.weforum.org/publications/global-gender-gap-report-2025/

16
https://github.com/BFH-AMI/BIAS

17
https://fasttext.cc/

18
https://huggingface.co/google-bert/bert-base-german-cased

19
https://huggingface.co/dbmdz/bert-base-german-cased

20
https://fasttext.cc/

21
https://fasttext.cc/

22
https://huggingface.co/NbAiLab/nb-bert-base

23
https://fasttext.cc/

24
https://fasttext.cc/

25
https://huggingface.co/dbmdz/bert-base-turkish-cased

26 The intermediate results are available at https://doi.org/10.5281/zenodo.15650909, final results are forthcoming.

27 Data available at https://doi.org/10.5281/zenodo.15747946.

28 Consistent with ethical principles and fundamental rights Council of Europe (2022) European Commission (2025).

29 Aligned with the call for a broader “paradigm shift toward comprehensively trustworthy AI systems.” Li et al. (2023), which is made more pronounced with the EU AI Act Laux et al. (2024).

30 Intermediate results available at https://doi.org/10.5281/zenodo.17351948, more advanced analyses are ongoing and will be published in the future.

31
https://github.com/BFH-AMI/BIAS
